# HER2-directed antibodies, affibodies and nanobodies as drug-delivery vehicles in breast cancer with a specific focus on radioimmunotherapy and radioimmunoimaging

**DOI:** 10.1007/s00259-020-05094-1

**Published:** 2020-11-12

**Authors:** Betül Altunay, Agnieszka Morgenroth, Mohsen Beheshti, Andreas Vogg, Nicholas C. L. Wong, Hong Hoi Ting, Hans-Jürgen Biersack, Elmar Stickeler, Felix M. Mottaghy

**Affiliations:** 1 Department of Nuclear Medicine, University Hospital Aachen, RWTH Aachen University, 52074 Aachen, Germany; 2 Center of Integrated Oncology (CIO), Universities of Aachen, Bonn, Cologne and Düsseldorf, Kerpener Str. 62, 50937 Cologne, Germany; 3 Division of Molecular PET-Imaging and Theranostics , Paracelsus Medical University , Salzburg, 5020 Austria; 4 Nanomab Technology Limited, Shanghai, People’s Republic of China; 5 Department of Nuclear Medicine, University Hospital Bonn, Bonn, Germany; 6 Department of Gynecology and Obstetrics, RWTH Aachen, Aachen, Germany; 7 Department of Radiology and Nuclear Medicine, Maastricht University Medical Center (MUMC+), 6202 Maastricht, The Netherlands

**Keywords:** HER2, Antibody drug conjugate, Nanobody, Single domain antibody, Affibody, Immunotherapy

## Abstract

**Purpose:**

The aim of the present paper is to review the role of HER2 antibodies, affibodies and nanobodies as vehicles for imaging and therapy approaches in breast cancer, including a detailed look at recent clinical data from antibody drug conjugates and nanobodies as well as affibodies that are currently under development.

**Results:**

Clinical and preclinical studies have shown that the use of monoclonal antibodies in molecular imaging is impaired by slow blood clearance, associated with slow and low tumor uptake and with limited tumor penetration potential. Antibody fragments, such as nanobodies, on the other hand, can be radiolabelled with short-lived radioisotopes and provide high-contrast images within a few hours after injection, allowing early diagnosis and reduced radiation exposure of patients. Even in therapy, the small radioactively labeled nanobodies prove to be superior to radioactively labeled monoclonal antibodies due to their higher specificity and their ability to penetrate the tumor.

**Conclusion:**

While monoclonal antibodies are well established drug delivery vehicles, the current literature on molecular imaging supports the notion that antibody fragments, such as affibodies or nanobodies, might be superior in this approach.

## Introduction

Breast cancer is the second most common cancer worldwide and the most frequent among women with an estimated 2.09 million new cases diagnosed in 2018 (11.6% of all cancers). It is the fourth cause of death from cancer overall and the leading cause of cancer death in women [1].

**Fig. 1:**
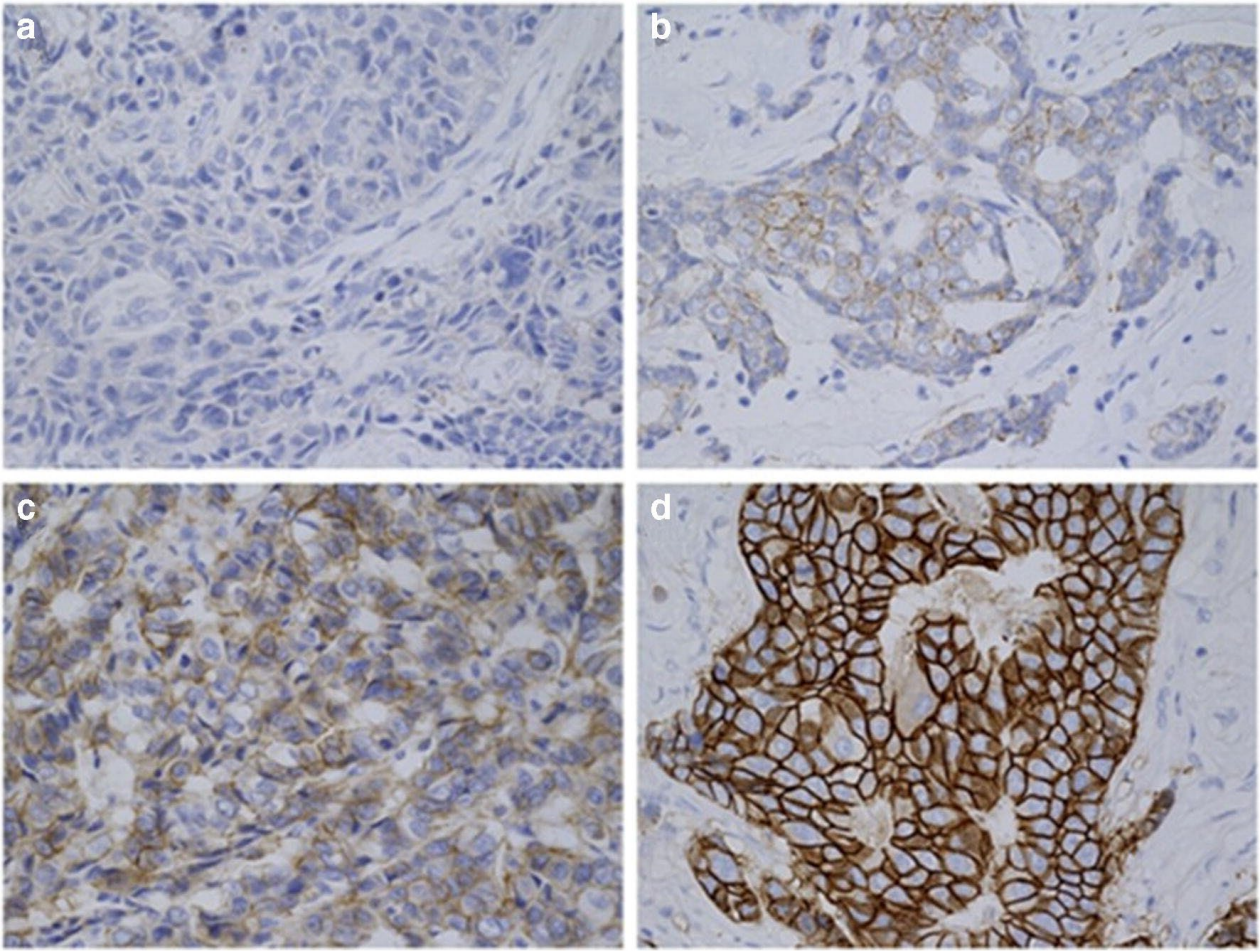
HER2 (human epidermal growth factor receptor 2) expression status determined by immunohistochemistry (IHC). Depicted are tissues from patients with invasive breast cancer (400x) whose HER2 status was determined by IHC. **a** Negative (score 0), **b** negative (score 1+), **c** equivocal (score 2+), **d** positive (score 3+). [6]

The general subtyping of breast cancer is based on the presence of transmembrane and intracellular receptors, namely, estrogen (ER), progesterone (PR) and the human epidermal growth factor receptor 2 (HER2, also referred to as ERBB2) [2, 3]. While reviews from previous years reported that approximately 25–30% of breast carcinomas show an overexpression of the oncoprotein HER2, the IQTIG sets the rate for Germany in 2019 at about 13%. The overexpression is due to the 2- to greater than 20-fold amplification of the protooncogene c-erbB2 [4, 5]. According to the guidelines of the American Society of Clinical Oncology (ASCO)/College of American Pathologists (CAP), the HER2 status can be divided into four categories by using immunohistochemistry (Fig. 1).

If no cells are stained or only a weak, barely perceptible membrane staining is present, the status is referred to as HER2 negative (score 0 and 1+). Score 2+, means an equivocal status, applies if a weak to moderate complete membrane staining can be observed in more than 10% of tumor cells. HER2 positive (score 3+) is defined as a complete, intensive staining of the circumferential membrane that occurs in more than 10% of tumor cells [7].

Several studies showed that the amplification of this tumor-associated antigen has a direct role in the pathogenesis of cancer [8–11]. This is because the HER2 receptor is activated by homo-/heterodimerisation and consequently triggers many important downstream signals, including the Mitogen-activated protein kinase (MAPK) and phosphoinositide 3-kinase (PI3K) signalling pathways. The signalling cascades recruit and regulate various proteins that, among other biological and clinical parameters, stimulate cell proliferation and survival. However, if the HER2 receptor is overexpressed, the cell cycle is disrupted and tumorigenesis is promoted [12]. Therefore, the higher HER2 is expressed, the lower the disease-free survival, the higher the risk of metastases and the shorter the overall survival (OS) rate [13, 14].

The introduction of monoclonal antibodies against the extracellular domain of the HER2 protein was considered a breakthrough in breast cancer therapy. The antitumor efficacy of HER2-directed antibodies is attributed besides the blockade of the HER2 pathway to the broad activation of the immune system, which leads to antibody-dependent cellular cytotoxicity [15]. Trastuzumab was the first humanized monoclonal antibody approved by the Food and Drug Administration (FDA) of the USA in 1998 and two years later by the European Medicines Agency (EMA) for the treatment of both early stage and metastatic HER2 overexpressing breast cancer [13, 16, 17]. In the clinic, trastuzumab is always combined with standard chemotherapy as a starting treatment in the neoadjuvant, adjuvant and metastatic setting, respectively. In all clinical situations, the outcome for patients cotreatment of trastuzumab was dramatically improved with reduced recurrence and improved disease-free survival rates but also improved overall survival in the metastatic situation (median survival, 25.1 vs. 20.3 months)[18–21]. The antibody’s remarkable activity and the favourable cytotoxicity profile achieved by its high binding specificity made it a favourable vehicle for carriers of other, less specific anti-cancer drugs [22, 23].

## Antibody drug conjugates targeting HER2

**Fig. 2:**
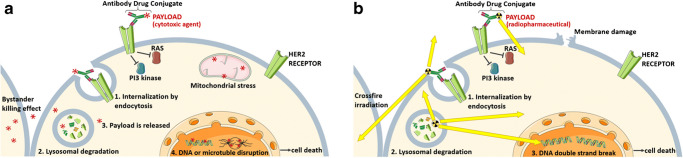
Mode of action of HER2-directed antibody drug conjugates with a **a** cytotoxic agent and **b** radiopharmaceutical payload. By binding of the antibody conjugate, the activation of the receptor and thus the intracellular signalling cascade is inhibited. After internalization and lysosomal degradation of the antibody receptor complex, the payload is released in the cytoplasm where it exerts its effect

The goal in the development of antibody drug conjugates (ADC) is to achieve increased cytotoxicity in the target cells while reducing chemotherapy off-target adverse events. ADCs are monoclonal antibodies covalently bound to a cytotoxic agent (called drug payload or warheads) by a synthetic linker. Thus, ADC’s combine the effector functions of antibodies in binding a specific target with the cytotoxic potency of a chemotherapeutic drug [24]. Important factors that define the success of ADCs are the selection of the payload and the characteristics of the linker conjugation, because they affect the stability, efficacy, pharmacokinetics, homogeneity and biophysical integrity of the conjugates. An ideal ADC payload is a highly potent small molecule with lack of specificity [25]. ADCs affect not only cancer cells expressing the antigen but also surrounding cells, a so-called bystander killing effect. This ADC approach is feasible by using cleavable linkers, which after (1) ADCs binding, (2) internalization by endocytosis and (3) transport to the lysosome, are cleaved releasing the cytotoxic payload. The free payload can then bind to its target within the cancer cell or diffuse into the intercellular space, due to their high membrane permeability, thus inducing cell death in neighbouring cells. Alternatively, the ADC-payload conjugate with a diffusible drug can be cleaved by extracellular enzymes prior to internalization of a diffusible drug (Fig. 2a) [26].

Besides monoclonal antibodies, antibody fragments can also be used as vehicles for ADCs. Antibody fragments such as minibodies, diabodies, single chain fragments of variable regions (scFvs) and nanobodies are parts of antibodies, modified through genetic engineering. They usually contain only the basic targeting and binding domain of antibodies. Due to their relatively small molecular weight (7–100 kDa) and low complexity, the antibody fragments exhibit better pharmacokinetics for non-invasive targeted imaging. In addition, their properties such as shorter circulation times, deeper tumor penetration and high specificity to the target make them preferable to monoclonal antibodies as vehicles for ADCs [27]. Affibody molecules are one of the most important engineered proteins for molecular imaging. The small antigen-binding domain is derived from Staphylococcal Protein A (SPA) and has a molecular weight of 6–7 kDa. Other much-researched antigen binding domains are nanobodies, which represent an antibody fragment consisting of a single monomeric variable antibody domain and are characterised by their low molecular weight of approximately 15 kDa and their fast blood clearance [28].

Currently, several ADCs targeting HER2 are under clinical investigation for breast cancer treatment.

### HER2 targeting monoclonal antibodies conjugated to a chemotherapeutic agent

Ado-trastuzumab emtansine (T-DM1, KADCYLA®) is the first EMA and FDA-approved ADC which targets HER2. It consists of trastuzumab connected to 3.5 molecules of DM-1 (mertansine or emtansine, derivatives of maytansine and potent microtubule inhibitors) by a non-cleavable linker [25, 29]. A phase I study published in 2010 was the first study demonstrating the safety and tolerability of T-DM1 [30]. In subsequent randomized phase III studies, the efficacy of this ADC therapy as adjuvant, neoadjuvant, first-line and second-line therapy in HER2-positive breast cancer was evaluated. The outcome of the TH3RESA (NCT01419197) and EMILIA study (NCT00829166) in patients with HER2-positive metastatic breast cancer previously treated with trastuzumab and a taxane demonstrated improvements in median OS (30.9 months vs. 25.1 months) and progression-free survival (PFS) (9.6 months vs. 6.4 months) in the T-DM1-treated group compared with lapatinib and capecitabine treated groups [31, 32]. On the other hand, the low efficacy of ADC in combination with pertuzumab in the MARIANNE and KRISTINE studies [33] led to the discontinuation of the KAITLIN study (NCT01966471), which was designed to evaluate the efficacy and safety of T-DM1 in combination with pertuzumab and a taxane as adjuvant therapy after anthracycline-based chemotherapy in participants with HER2-positive primary invasive breast cancer. A meta-analysis reported that the most common adverse events of all-grade in patients receiving T-DM1 include fatigue, nausea, musculoskeletal pain, hemorrhage, thrombocytopenia, headache, increased transaminases, constipation and epistaxis. The main toxicities of T-DM1 are considered to be caused by the payload, but further research is needed [34]. Most of these adverse events are generally of low grade and manageable, except for severe thrombocytopenia (grade ≥ 3). For this reason, patients with severe cardiac dysfunction, increased liver enzymes, or in cases of severe thrombocytopenia should have their dose adjusted or treatment with T-DM1 discontinued [35, 36].

Another recently approved ADC is trastuzumab deruxtecan (DS8201, Enhertu®), which is a humanized trastuzumab antibody conjugated with a topoisomerase I inhibitor (DXd, an exatecan derivative). The drug to antibody ratio (DAR) is approximately eight higher than in all currently approved ADCs [37, 38]. In preclinical models, DS8201 was shown to be well tolerated and able to overcome T-DM1 resistance [25, 39, 40]. A major advantage of this ADC is that it is effective at different levels of HER2 expression [41]. FDA approval of this ADC was granted after demonstrating antitumor efficacy and safety in a phase II dose-finding study (DESTINY-Breast01, NCT03248492) in patients with HER2-positive, unresectable and/or metastatic breast cancer after two or more anti-HER2 therapy cycles [42]. Currently, eight registered ongoing trials investigating DS8201 are recruiting (NCT04042701, NCT03523572, NCT03505710, NCT04132960, NCT04014075, NCT03523585, NCT03529110, NCT03734029). The results of these studies are unpublished yet, but based on the findings of previous studies, treatment with DS8201 could be a valuable therapy option with the potential to address the T-DM1 insensitive breast cancer and other HER2 expressing cancers.

Other ADCs consisting of a monoclonal antibody and a cytotoxic agent, which are currently being investigated in clinical trials, are summarized in Table 1.

**Table 1:** Overview of human trials of HER2 targeting non-approved immunotherapeutic conjugates, their composition and their current state of development. Status of April 2020

ADC	Antibody	Payload	Trial no.	Phase	Patients	First posted	Status
BAT8001	Trastuzumab	Maytansine derivative	NCT04189211	I	30	12/2019	Active, not recruiting
			NCT04151329	I / II	72	11/2019	Enrolling by invitation
			NCT04185649	III	410	12/2019	Active, not recruiting
[vic-] Trastuzumab Duocarmycin (SYD985)	Trastuzumab	vc-*seco*-DUBA	NCT02277717	I	185	10/2014	Completed
			NCT04235101	I	120	01/2020	Recruiting
			NCT04205630	II	60	12/2019	Recruiting
			NCT03262935	III	345	08/2017	Recruiting
Hertuzumab Vedotin (RC-48)	Hertuzumab	Monomethylauristatin E	NCT02881190	I	57	08/2016	Completed
			NCT02881138	I	50	08/2016	Recruiting
			NCT04311034	I	36	03/2020	Recruiting
			NCT03052634	I / II	90	02/2017	Recruiting
			NCT04264936	I / II	36	02/2020	Recruiting
			NCT04329429	II	57	04/2020	Recruiting
			NCT03809013	II	60	01/2019	Recruiting
			NCT04073602	II	18	08/2019	Recruiting
			NCT03556345	II	127	06/2018	Active, not recruiting
			NCT03500380	II	228	04/2018	Recruiting
MM-302	PEGylated antibody	Liposomal doxorubicin	NCT01304797	I	75	02/2011	Unknown
			NCT02213744	II / III	113	08/2014	Terminated
ARX788	Anti HER2 antibody	Amberstatin269	NCT03255070	I	60	08/2017	Recruiting
XMT-1522	HT-19	Auristatin F-hydroxypropylamide	NCT02952729	I	120	11/2016	Active, not recruiting
MEDI4276	bi-paratopic antibody	AZ13599185	NCT02576548	I / II	47	10/2015	Completed
DHES0815A	Trastuzumab derivative	pyrrolobenzodiazepine	NCT03451162	I	14	03/2018	Active, not recruiting
BDC-1001	Trastuzumab	TLR7/8 agonist	NCT04278144	I	390	02/2020	Recruiting
ALT-P7 (HM2-MMAE)	HM2	monomethylauristatin E	NCT03281824	I	30	09/2017	Recruiting
ADCT-502	Trastuzumab	Tesirine	NCT03125200	I	21	04/2017	Terminated
PF-06804103	Anti HER2 antibody	Auristatin-0101	NCT03284723	I	124	09/2017	Recruiting

Several other HER2-targeting ADCs are currently undergoing preclinical trials for example LCB14-0110. This ADC is composed of monoclonal HER2 directed antibody trastuzumab linked via a beta-glucuronide linker to monomethylauristatin F (MMAF). However, no data have been published so far [43]. Another ADC which is currently investigated in preclinical studies is MI30004. This ADC consists of a humanized trastuzumab antibody connected by a noncleavable linker to two molecules of payload PM050489, which binds to β-tubulin with very high affinity and disrupts the microtubule network, resulting in mitotic aberrations and cell death. In vitro and in vivo analyses of MI130004 in different tumor cell lines, including breast, ovarian and gastric cancer, showed that MI130004 generated a long-lasting antitumor effect with a statistically significant inhibition of tumor growth and increased the median survival time compared to treatment with trastuzumab. Its therapeutic efficacy still has to be evaluated in a clinical trial [44].

### Radiolabeled HER2 targeting monoclonal antibodies

Radiopharmaceuticals can also be used as a payload in ADCs. The intravenous or intratumoral injection of a monoclonal antibody tightly labeled with a radionuclide is called radioimmunotherapy or immunoimaging, depending on the purpose. The specific binding of the antibodies to their target allows a direct transport of the radionuclide to the tumor and leads to cell death through radiation-induced double strand DNA breaks and the formation of reactive oxygen species in the case of beta-, alpha or Auger electron-emitters (Fig. 2b) or enables targeted molecular imaging (immunoimaging). The efficacy of radioimmunotherapy depends on the radiation quality or linear energy transfer (LET), which refers to the amount of deposited energy per unit track length. The β-emitters produce a low LET radiation of about 0.2 keV/μm, release energies of 30 keV and 2.3 MeV and have a long range within the tissue (0.5–12 mm) thus causing a crossfire effect. α-emitters, on the other hand, can produce high LET radiation of 50–230 keV/μm with energies of 5 to 9 MeV, but have a much shorter range in tissue (50–100 μm). This reduces the toxicity of α-emitters compared to β-emitters to neighbouring cells and increases the number of ionisations per emission. Auger electron emitters are characterised by a medium LET radiation (4–26 keV/μm) with energies between 1 eV and 1 keV and a range in tissue of less than 1 μm, but a high emission density. This results in an intensive energy deposition within a nanometer range, thus requiring the deposition of Auger electron radiation to the cell DNA. The therapeutic effect is achieved by inducing severe DNA damage [45, 46]. Due to the different properties of the emitters, the effectiveness of radioimmunotherapy to a large extent depends on the selection of the isotope. By applying other radionuclides with comparable chemical properties the same labeling precursor or radiopharmaceutical can be used for both, molecular imaging and therapy, which represents the concept of theranostics [47]. For molecular imaging, gamma emitting or positron-emitting radionuclides are applied for single photon emission computed tomography (SPECT) or positron emission tomography (PET) respectively [48]. For both, radiolabeling and purification purposes by immobilised metal affinity chromatography, a C-terminal amino acid tag can be inserted genetically into the antibody or its fragment [49]. Depending on the chosen radionuclide, radiolabeling of antibodies requires an additional chelating agent for complexation or a prosthetic group for electrophilic substitution. Several conjugation strategies have been described therefore [50–53]. It shall not be underestimated, that the type and placement of the chelator can influence the tumor-targeting properties, the blood clearance rate and uptake into healthy tissue of the antibody [54]. By enabling early detection, real-time therapeutic monitoring and the ability to streamline drug development, molecular imaging is preferable to invasive tissue sampling, which is usually limited to a single time point and cannot capture tumor heterogeneity [55].

Up to date, numerous investigations with radiolabeled monoclonal antibodies that address HER2 have been conducted. The positron emitting zirconium-89 labeled trastuzumab is one of the most investigated. For this purpose the radiometal is linked to the antibody via the chelator DFO (deferoxamine). Although animal studies have shown that this chelator is not stable leading to the release of the radiometal during circulation and it’s accumulation in bones (15–20% injected dose per gram [ID/g]) [56], this phenomenon has not been observed in clinical studies [57]. Nevertheless, several attempts have been made to find a new chelator. However, none of the tested chelators L1-L4, which are based on hydroxamate-functionalized macrocycles, showed improved in vivo stability [58, 59]. Nevertheless, already in the first clinical trial with ^89^Zr-Df-Bz-NCS-trastuzumab in patients with HER2-positive metastatic breast cancer, a high tumor uptake (33.4 ± 7.6% ID/g) including a depiction of metastases was achieved [60]. Two other clinical trials (NCT01832051, NCT01565200) have shown the potential of imaging HER2 with ^89^Zr-trastuzumab. While one study demonstrated that ^89^Zr-trastuzumab supports clinical decision making when HER2 status could not be determined by standard procedures (bone scan, ^18^F-FDG PET, CT and biopsy), the other study was able to determine tumor heterogeneity. This allows the selection of a personalized therapy [61, 62]. In a further phase I clinical trial (NCT02065609) the liver was determined as the dose-limiting organ at a dose of 1.63 mSv/MBq. Since only slow blood clearance with a biological half-life of 113 h and an initial level of 58% ID in the blood pool was observed, imaging with ^89^Zr-trastuzumab was considered safe with acceptable but not satisfactory dosimetry [63]. In another phase I clinical trial (UMIN000004170), the copper-64 labeled ADC, ^64^Cu-DOTA-trastuzumab, proved to be safe and effective in identifying HER2-positive lesions in patients with primary and metastatic breast cancer when high liver uptake was reduced by administration of 45 mg cold trastuzumab. Although the HER2 specificity was confirmed by autoradiography, immunohistochemistry scores and LC-MS/MS, the relationship between HER2 PET imaging and the effects of anti-HER2 therapy still need to be evaluated [64–66]. Bhusari et al. were able to show the safety of lutetium-177 labeled trastuzumab, as a radioimmunoconjugate, in a phase I clinical trial. The authors reported also specific tracer uptake in the HER2-positive primary and metastatic breast lesions. Due to a high uptake the liver is considered to be the dose-limiting organ (tumor to background ratio of 0.38 on day 1). ^177^Lu-trastuzumab may be used for palliative purpose in combination with other conventional treatments for HER2-positive metastatic breast cancer, but further clinical trials with escalating antibody doses and dosimetric evaluation are needed [67].

In the area of radioactively labeled ADCs, an attempt is made to label the antibody trastuzumab with other radioisotopes, such as indium-111 [68–70], technetium-99m [71], rhenium-188 [72–75], thorium-227 [76] or iodine-131 [77]. In a preclinical study for example, Li et al. were able to show that ^111^In-trastuzumab-NLS (Nuclear Localizing Signal) can modulate the NF-κB signalling pathway. They also showed that the coinjection of bortezomib can inhibit the growth of HER2 overexpressing breast cancer SK-BR-3 cells [68]. In another study, the ^111^In-trastuzumab was linked to gold nanoparticles (AuNP). These specifically bound to HER2 positive SK-BR-3 cells and caused lethal DNA double-strand breaks. In mice with subcutaneous HER2-positive breast cancer xenografts, an intratumoral injection of trastuzumab AuNP-^111^In inhibited tumor growth without obvious normal tissue toxicity [69]. To our knowledge, there is no clinical study comparing radiolabeled trastuzumab with unlabeled trastuzumab. However, preclinical studies have shown that a 5-fold increase in toxicity of ^177^Lu-DOTA-trastuzumab compared to unlabeled trastuzumab was observed in SK-BR-3 cells (relative number of survived cells after 120 h 10 ± 3.5 % vs. 41 ± 2.8 %) [78]. In two further studies, the higher cytotoxic potency of ^111^In labeled trastuzumab derivatives—making use of the therapeutic Auger electron emission—compared to unlabeled trastuzumab was shown in vitro. While ^111^In-NLS_6_-trastuzumab was 6 times more effective at killing SK-BR-3 cells than the cold antibody (relative number of survived cells 10.5 ± 2.1 % vs. 64.6 ± 3.0 %) [79], the administration of ^111^In-Hy-MCP trastuzumab with a high specific activity showed a 20.5-fold higher cytotoxic potency (relative number of survived cells 1.8 ± 1.3% vs. 37.0 ± 5.3%) [80]. This shows that radioactively labeled antibodies can be more effective in treating tumors than unlabeled ones and that further research in this area is needed.

Further research is also conducted on the radiolabeling of the antibody pertuzumab. Preclinical studies have shown that ^64^Cu-NOTA pertuzumab is a good PET tracer that specifically targets HER2 receptors in breast cancer xenografts in NOD/SCID mice [81, 82]. This could also be demonstrated for ^89^Zr-pertuzumab [83]. In addition, Marquez et al. show for this radioconjugate that increased tumor uptake also occurs when co-injected with trastuzumab [83]. In the field of radioimmuntherapy ^177^Lu-pertuzumab was investigated. Persson et al. demonstrated the good intracellular retention of the radiolabeled antibody, both in vitro and in vivo, and its HER2 specific binding [84, 85].

### Disadvantages of HER2 addressing therapeutic monoclonal antibodies

Monoclonal antibodies such as trastuzumab or pertuzumab are often used as vehicles for the specific administration of a therapeutic drug to its target due to their specificity and affinity for their antigen. Even though their use in breast cancer therapy was initially very successful, there are still insurmountable limitations associated with their use in targeted therapy. The relatively high molecular weight (~ 160 kDa) of antibodies, the heterogeneous blood perfusion, the hindered diffusion in the interstitium, the extravascular binding of monoclonal antibodies and the increased interstitial pressure (turgor effect) leads to a heterogeneous distribution of the antibodies in the tumor. Also, due to their large molecular size, antibodies cannot be filtered by the kidney and accumulate in the liver, leading to hepatotoxicity [86–88]. In addition, it has been shown that the high affinity of the antibodies impedes homogeneous tumor penetration and intratumoral diffusion, as the agent can get stuck at the periphery [89, 90]. This incomplete tumor penetration leads to a suboptimal therapeutic efficiency, which is one of the reasons for the development of resistance to antibody-based therapy [91]. Another disadvantage is slow blood clearance of monoclonal antibodies that lasts between few days and weeks, whereby good contrast images, which are achieved by a high tumor to background ratio, can only be obtained after hours or days after application. For this reason, radiolabeling with long-lived radionuclides is necessary. Moreover, monoclonal antibodies show a considerable degree of non-specific uptake at the target sites, especially at the earlier time-points, and can only be administered intravenously or subcutaneously due to their low thermodynamic stability [60, 92, 93]. For ADCs to activate their antibody-dependent cell-mediated cytotoxicity (ADCC), it is desirable that the antibody-antigen complex is not rapidly internalized. However, phagocytosis is mediated by the Fc region of the antibody, thereby reducing the availability of the antibody on the cell surface to unfold its desired mechanism of action [94, 95]. In addition, only few antibodies are able to cross the blood-brain barrier and reach the central nervous system, making it difficult to detect and treat brain metastases. These limitations have initiated and driven the development of smaller antibody fragments as vehicles with better tissue penetration and higher cytotoxic efficacy [96].

## Affibodies

Affibodies (Affibody®) are technically produced antibody fragments that can be used as theranostic tools. Affibodies derived from Staphylococcal surface protein A form a cysteine-free three-helix scaffold protein consisting of 58 amino acids and contain no disulfide bridges, thus increasing the stability of the molecule. Due to their high affinity and tolerance to chemicals, higher temperatures and extreme pH values, as well as their small size and short circulation time, affibody molecules are very well suited for a use in molecular imaging [97].

After preclinical studies [98–101] showed successful tumor targeting and imaging for HER2-directed affibody molecules, they were further investigated in clinical trials (Table 2). The results of the first clinical trial with the radiolabeled HER2-targeting affibody ABY-002 (DOTA ZHER2:342 pep2) in patients with recurrent breast cancer were very promising (EudraCT 2007 002530 11). With the indium-111- and gallium-68 labeled ABY-002, high-quality SPECT and PET images, respectively, could be acquired after only 2 h post-injection (p.i.). The majority of lesions previously detected with ^18^F-FDG-PET could be confirmed with the radioactive affibody. Only those near the kidney and liver could not be detected due to the high background uptake [102]. Therefore, this tracer was further modified to achieve a better blood clearance and a higher background to tumor ratio. Another affibody that has been investigated in a clinical trial is ABY-025 (ZHER2:2891) (NCT01216033). The ^111^In-labeled affibody demonstrated favourable biodistribution, safety, dosimetry and tumor targeting potential in patients with HER2-positive metastatic breast cancer. In addition, high-contrast SPECT images were obtained within 4 to 24 h p.i., although the highest uptake in normal tissue was in the kidneys, followed by the liver and spleen [103]. In two further clinical studies the same affibody was examined with a ^68^Ga label (NCT02095210, NCT01858116). By administering two different doses of peptide (100 μg or 500 μg) the effects on the uptake in tumors were investigated. PET images after 2 to 4 h p.i. showed that injection of 500 μg ^68^Ga-ABY-025 led to better specificity and allowed differentiation between metastases with the HER2 expression levels of score 3+ and score 2+ [104, 105]. ^68^Ga-ABY-025 is currently being investigated in a phase II/III clinical trial to determine the correlation between HER2 expression measured with ^68^Ga-ABY-025 PET and standard histopathology from relevant tumor biopsies (NCT03655353). Administration of ^99m^Tc-labeled HER2-targeting affibody ABH2 in an open-label phase I clinical trial (NCT03546478) in HER2-positive breast cancer patients showed a specific binding (overall specificity 60%) of the affibodies to their target molecule without noticeable adverse effects for the patient. After only 1.5 and 4.5 h p.i., high-contrast SPECT images were obtained, but the uptake of the radiotracer by the liver was so high (T/B ratio = 21.9 ± 3.5), that HER2-positive liver metastases could not be detected [106]. Another ^99m^Tc-labeled affibody (HPArk2) is currently being investigated in an open label phase I clinical trial, but so far no results have been published (NCT04267900). In a further open label, non-randomised clinical trial, the efficacy of [^18^F]GE-226 in determining HER2 expression level in patients with metastatic breast cancer is being investigated (NCT03827317). Furthermore, the pharmacokinetics of the affibody and the optimal time for the PET scan will be determined. However, no results have yet been reported for this study.

**Table 2:** Overview of human trials of HER2 targeting affibodies in breast cancer patients. Status of April 2020

Affibody	Radioisotope	Diagnostic/therapy	Trial no.	Phase	Patients	First posted	Reference
ABY-002	^68^Ga	PET	EudraCT 2007 002530 11	Pilot study	3	07/2007	[102]
	^111^In	SPECT					
ABY-025	^111^In	SPECT	NCT01216033	I/II	7	10/2010	[103]
	^68^Ga	PET	NCT02095210	I	8	03/2014	[104]
			NCT01858116	I/II	16	05/2013	[105]
			NCT03655353	II/III	120	08/2018	Nonpublished
ABH2	^99m^Tc	SPECT	NCT03546478	I	32	06/2018	[106]
HPArk2			NCT04267900	I	30	02/2020	Nonpublished
GE-226	^18^F	PET	NCT03827317	Not applicable	16	02/2019	Nonpublished

Despite the numerous advantages and features that make affibodies particularly suitable for molecular imaging, there are still some hurdles to overcome. For example, the low affinity of affibodies to the target is a major issue [27]. In addition, the design of the affibody molecules would have to be modified in order to reduce off-target interactions or background radioactivity [107]. However, the development of radioactively labeled affibodies is expensive and poses some challenges in scaling-up of the production process [27]. Moreover, the labeling approaches can lead to increased lipophilicity, which often leads to off-target interactions with normal tissue and binding to blood proteins [107]. A further disadvantage could be the bacterial origin of the protein scaffolds, as the risk of immunogenicity is increased after repeated therapeutic administration to patients [108]. Further clinical studies will be necessary to optimise the dose, time, sensitivity and specificity of these ligands, but also to improve the therapeutic application, which has so far been hampered by the short retention time of the affibody molecules in the blood.

## Nanobodies

**Fig. 3:**
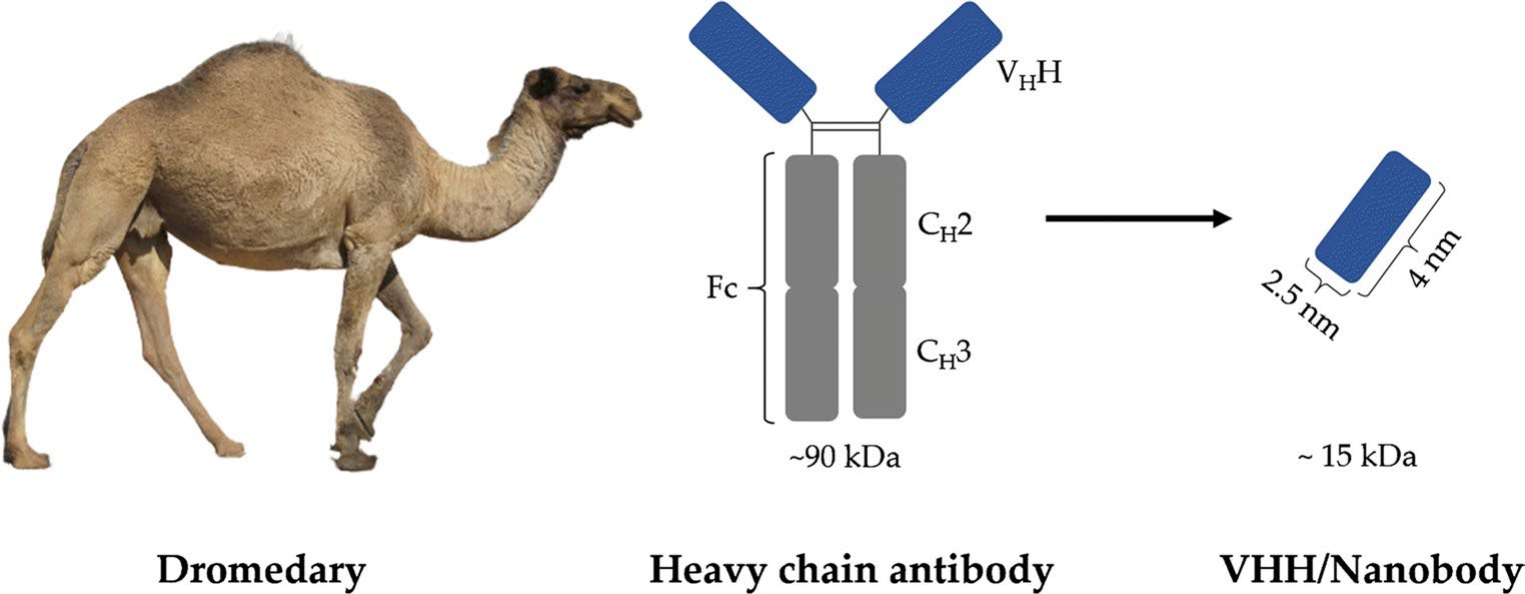
Schematic representation of a heavy chain antibody of dromedaries. Each variable domain (VHH) of the HcAbs is connected to a hinge domain and further to CH2 and CH3 domains. The CH2 and CH3 domains form the Fc domain. The VHH domain represents the smallest intact functional antigen-binding region of HcAbs and is also called nanobody

Most antibodies are Y-shaped and are composed of two heavy and two light polypeptide chains. In addition to these conventional antibodies, camelid species (i.e. Camelus dromedarius, Camelus bactrianus, Lama glama, Lama guanoco, Lama alpaca and Lama vicugna) and sharks produce heavy chain antibodies (HcAb, cf. Fig. 3) [109, 110]. Since the light chain is missing, the HcAbs bind to their antigen only by a single variable domain that is directly linked to the Fc domain (CH2 and CH3) via a hinge region. The variable domain is called VHH in camelids and VNAR in sharks [111, 112]. The VHH in an HcAb is the structural and functional equivalent of the Fab fragment of conventional antibodies and is generally referred to as Nanobody™ or single-domain antibody (sdAb) due to its low molecular mass of only 15 kDa (Fig. 3) [113]. The low molecular weight offers the advantage that the nanobodies can be eliminated via the kidney, which makes their biological half-life very short.

The crystal structures of several nanobodies showed that the scaffold of the VH and the VHH are identical. The scaffold of the prolate particle VHH with 2.5 nm diameter and about 4 nm height, consists of nine antiparallel β-strands forming two β-sheets stabilized by a conserved disulfide bridge [112, 114, 115]. Minor differences between the complementarity determining regions (CDR) of VH and VHHs explain for the strong antigen binding capacity of the camel-derived nanobodies [112]. For example, the CDR3 region of nanobodies is on average longer than that of VH and can be stabilized by an additional disulfide bond that connects the CDR3 to the adjacent CDR1 loop (common in VHH and VNAR) or to the CDR2 loop (common in Lama sdAbs) [116]. The elongated CDR3 region can form finger-like extensions that can extend into small cavities on the surface of the antigens which compensates for the absence of three other antigen-recognizing CDRs located in the light chain of conventional antibodies [117]. In addition, the hydrophobic to hydrophilic amino acid substitutions in the CDR2 region result in a structure with improved water solubility that is less susceptible to aggregation [116, 118]. Despite these differences, nanobodies exhibit a high degree of sequence homology with the VH and are therefore considered to have a low immunogenic profile [119]. Nevertheless, the nanobodies can be further humanized by simple site-directed mutagenesis to reduce a possible immune response [120].

Nanobodies have many technological and biophysical advantages, making them superior to conventional antibodies in several areas. In addition to the high water solubility mentioned above, nanobodies are also very stable. Even after a 1-week incubation at 37 °C, three of four nanobodies tested showed a binding activity of 100% and one nanobody 80% [121]. Melting temperature was set at over 60 °C, and even at temperatures up to 90°C the nanobodies showed their antigen-binding specificity, indicating high thermal stability [122, 123]. A high resistance of nanobodies to denaturing chemicals (8 M urea, 3 M guanidinium hydrochloride) has also been demonstrated. Immediately after diluting the chaotropic solution in water, the completely denatured nanobody folded back into its native form, which creates the conditions for sanitising the nanobodies and regenerating them several times [122]. Even exposure to non-physiological pH and elevated pressure were not able to impact the antigen binding capacity of nanobodies [124].

To obtain nanobodies, camelids are immunised with the antigen of interest, the DNA or with cells that express the antigen on their surface. After screening the nanobodies can then be easily expressed in microorganisms (Escherichia coli, Saccharomyces cerevisiae and Pichia pastoris), mammalian cells and plants due to their monomeric structure and the absence of post-translational modifications [93, 125, 126]. Production and selection advantages, such as the scalability of the production process or the easy cultivation in shake flasks, lead to high expression yields at low production prices [115].

### Radiolabeled nanobodies

Due to their small size and high affinity, nanobodies are particularly suitable for penetrating tumor tissue and binding to the antigen with high specificity [127]. In order to use nanobodies as a theranostic tool, they must first be labeled with a suited radioactive nuclide. Since the biological half-life of the nanobodies is short, radionuclides with a short physical half-life can also be used. This would allow diagnostic scans to be taken just a few hours after tracer injection [128, 129].

Generally, due to the small size of nanobodies, an improved blood clearance compared to conventional antibodies could be verified: In several animal studies, it was shown that one hour after injection, less than 0.5% of the injected activity per gram tissue was present in the blood pool, resulting in a better signal-to-noise ratio for the specifically bound radiolabeled fragment and less toxic effects [92, 130, 131]. On the other hand, a rapid blood clearance could prevent the radiolabeled nanobody from circulating in the patient's blood and therefore only a small fraction of the administered nanobody reaches its target. Hence, multiple doses of the nanobody should be administered to achieve a high target load in vivo. The main disadvantage of using radiolabeled nanobodies as in vivo imaging probes is their accumulation in the kidneys, which is a consequence of their renal elimination. Due to their small size, which is below the renal threshold for glomerular filtration, the nanobodies are reabsorbed by the proximal tubules through the key endocytic receptor megalin so that they remain in the renal cortex. For this reason, nephrotoxicity can occur in renal cells due to the radiation dose. Also, the sensitivity for the detection of a specific molecular signal in the vicinity of the kidneys, such as in the pancreas, is severely limited [132, 133]. Tchouate Gainkam et al. showed that the renal retention of the radiolabeled anti-EGFR nanobody (^99m^Tc-7C12) can be reduced by 36% by coinfusion from the plasma expander gelofusin [133, 134]. Gelofusin is a succinylated gelatin and increases the urinary excretion of proteins, especially those of low molecular weight [135]. A reduction of renal retention by about 45% was observed due to the additive effect of coinfusion of lysine and gelofusin with the radiolabeled nanobody ^99m^Tc-7C12 [132, 133]. In addition, removal of the amino acid tag (His6) at the C-terminus can further reduce kidney retention and help to prevent immunogenic reactions [131, 136, 137]. However, apart from the accumulation of radiolabeled nanobodies in kidney and urine, biodistribution is antigen-specific, resulting in a high tumor to background ratio early after administration, allowing subsequent diagnostic scans [138, 139].

#### Radiolabeled nanobodies in clinical studies (diagnostic and therapeutic approaches)

Keyaerts et al. conducted the first clinical study with a radioactively labeled nanobody, the ^68^Ga-NOTA-2Rs15d (EudraCT 012001135-31) [140]. The nanobody 2Rs15d was identified by screening using technetium-99m label as the best nanobody for imaging HER2-positive tumors that does not interfere with the therapeutic agent trastuzumab [92]. The PET nuclide ^68^Ga was chosen because it is cyclotron independent, nuclide generator based and with its short half-life of 68 min is suitable for use in humans. The NOTA derivative p-SCN-Bn-NOTA was applied as conjugated chelator enabling a fast and efficient ^68^Ga radiolabeling at room temperature while its in vivo stability was high [131]. The results of the first clinical phase showed a favourable biodistribution with a high uptake of the tracer in the tumor (standardized uptake value 0.7–11.8). Furthermore, rapid blood clearance was observed, with only 10% of the injected activity (IA) remaining in the blood 1 h after injection. In addition, a high tumor to background ratio was detected except for the kidney, liver and intestine regions. The optimal time for imaging was determined as 90 min after injection of the radiolabeled nanobody. The effective dose was 0.043 mSv/MBq. No symptoms or signs of toxicity were observed after administration of ^68^Ga anti-HER2 nanobody using 0.01–1 mg of nanobody per injection [140], which is why it is considered safe and is currently being investigated in an open label non-randomized monocenter phase II trial to evaluate its potential to detect brain metastases in breast cancer patients (EudraCT 2015-002328-24, NCT03331601) [141]. The correlation between image-based HER2 quantification after uptake of ^68^Ga-NOTA-2Rs15d in local or distant metastases of breast cancer patients and the results obtained by biopsy of the same lesion (NCT03924466) is under investigation in a further phase II clinical trial (VUBAR).

A phase I clinical trial (NCT04040686) is currently ongoing to evaluate the safety, dosimetry and efficacy of ^99m^Tc labeled anti-HER2 nanobodies in diagnostic imaging of HER2 in breast cancer patients. Subsequently, the results of molecular imaging will be compared with the results of HER2 expression by biopsy tissue immunohistochemistry and/or fluorescence in situ hybridization (FISH). The radionuclide ^99m^Tc is particularly suitable because it is available in almost every Nuclear Medicine unit via a generator system and the labeling process is simple and fast. In addition, the half-life (~ 6 h) fits to the fast blood clearance of nanobodies, allowing early diagnostic SPECT images with good contrast. This was proven in a preclinical study. Vaneycken et al. tested 38 different ^99m^Tc labeled nanobodies to find a lead compound. The nanobody ^99m^Tc-2Rs15d was found to be stable at least up to 24 h in PBS and serum and to interact specifically with the HER2 antigen. It also showed high tumor uptake (4.19 ± 0.47% IA/g at 1.5 h p.i.), rapid blood clearance, low accumulation in non-target organs other than the kidneys and a high tumor to background ratio (tumor-to-muscle ratio 49.6 ± 11.8, tumor-to-blood ratio 16.4 ± 3.6 at 1 h p.i.) [92, 142].

Copper-64 (half-life 12.7 h) is a hybrid beta emitter and has decay characteristics that allow for both, PET imaging and radioimmunotherapy. Lee et al. have investigated copper-64 radiolabeled MM-302 with simultaneous administration of trastuzumab regarding its enhanced permeability and retention effect in patients with HER2-positive metastatic breast cancer. The tumor accumulation of ^64^Cu-MM-302 after 24 to 48 h ranged from 0.52 to 18.5 %ID/kg and varied across lesions within a patient and between patients. Depositions in bone and brain lesions were also observed and a significant background uptake of ^64^Cu-MM-302 in liver and spleen. Presumably, the discrepancy in the results led to the discontinuation of the phase I clinical trial (NCT02735798) [143, 144].

Iodine-131 decays with a half-life of 8.02 days with beta- and gamma emissions and is used for diagnosis (SPECT) and especially therapy in Nuclear Medicine. One study investigated the radioiodination of the nanobody 5F7 via the residual prosthetic group SGMIB [145]. In a previous study it was shown that the residualizing agent is particularly suitable for achieving good tumor retention and short renal retention [146, 147]. However, since the radiolabeled nanobody 5F7 competes with trastuzumab for binding to domain IV on HER2, the same radiolabeling was tested with the nanobody 2Rs15d. In the preclinical study, ^131^I-SGMIB-2Rs15d was shown to specifically bind to HER2 on a different epitope than trastuzumab. Although tumor uptake was lower (20.22 ± 1.64% IA/g at 1 h p.i.) for ^131^I SGMIB 2Rs15d, a high tumor to background ratio, rapid blood clearance (< 2% IA/ total blood volume at 1 h p.i.) and short renal retention were observed. In addition, ^131^I-SGMIB-2Rs15d alone or in combination with trastuzumab was shown to significantly prolong median survival compared to animals treated with a ^131^I control nanobody (R3B23) (137.5 days vs. 93.5 days) [148]. A phase I clinical trial (NCT02683083) evaluated the safety, biodistribution and radiation dosimetry of ^131^I-SGMIB-2Rs15d in healthy volunteers and patients with HER2-positive breast cancer. Preliminary results showed a high tumor to background ratio, rapid blood clearance and elimination of unbound nanobodies via the kidney and no drug-related adverse events after intravenous administration (38 MBq ± 9 MBq). In addition, SPECT images showed that the nanobody was partially accumulated in metastases [149]. These promising results and favourable dosimetry would allow administration of therapeutic doses of ^131^I-SGMIB-2Rs15d with a minimum risk of radiotoxicity.

An overview of to date clinically evaluated radiolabeled nanobodies with potential application in breast cancer patients is provided in Table 3.

**Table 3:** Overview of human clinical trials of HER2 targeting radiolabeled nanobodies in breast cancer patients. Status April 2020

Nanobody	Radioisotope	Diagnostic/therapy	Trial no.	Phase	Patients	First posted	Outcome	Reference
2Rs15d	^68^Ga	PET	EudraCT 2012-001135-31	I	20	2012	Favourable biodistribution, high tumor to background ratio, fast blood clearance no signs of toxicity, urinary bladder as dose limiting organ	[140]
			EudraCT 2015-002328-24 NCT03331601	II	30	07/2015 11/2017	Ongoing	Nonpublished
			NCT03924466		20	04/2019	Ongoing	Nonpublished
	^131^I	SPECT & Therapy	NCT02683083	I	9	02/2016	A high tumor to background rate, fast blood clearance, no signs of toxicity	[149]
	^99m^Tc	SPECT	NCT04040686	I	10	08/2019	Ongoing	Nonpublished
MM-302	^64^Cu	PET and therapy	NCT02735798	I	0	04/2016	Tumor accumulation vary between and within patients, background uptake in liver and spleen withdrawn	[143]

#### Radiolabeled nanobodies in preclinical studies (diagnostic and therapeutic)

Because of its short half-life (~ 110 min) Fluor-18 is one of the preferred radionuclides for PET imaging. Thus, numerous ^18^F labeled nanobodies have been designed and evaluated as PET tracer. Despite of intensive studies, no useful HER2 targeting ^18^F labeled nanobody has been developed until now. In the first trials the HER2-targeting nanobodies 5F7 and 2Rs15d were labeled with the prosthetic group [^18^F]-SFB [150, 151]. Since the overall radiochemical yield of [^18^F]-SFB-2Rs15d was very low (5–15%) [150] and the nanobody 5F7 competes with trastuzumab for the HER2 binding site [152], the two nanobodies were labeled in another trial with [^18^F]-RL-I or [^18^F]-ADIBO via SPAAC (^18^F-RL-II-2Rs15d). With these tracers, excellent tumor targeting could be observed in HER2 positive cancer cells and xenotransplants, but the labeling procedure was too long and the radiolabeling yields were too low. In addition, unexpectedly high tracer accumulation in the liver, lung, spleen and kidney were observed [151–154]. Much better results were obtained in another study with the nanobody 2Rs15d which was labeled with [^18^F]-TFPFN and [^18^F]-AlF-NOTA-Tz-TCO-GK. The radiochemical yield and tumor to background ratios were high and the radiolabeled nanobody bound to the HER2 antigen with high affinity and high immunoreactivity. In addition, the renal uptake was reduced by more than 15-fold compared to [^18^F]-RL-II-2Rs15d and by about threefold compared to the level reported for [^18^F]-SFB-2Rs15d [155, 156].

Puttermans et al. have investigated ^111^In labeled 2Rs15d via p-SCN-Bn-CHX-A″-DTPA (DTPA*) as a theranostic radiopharmaceutical in breast cancer mediated brain metastases. In the trial, twenty-one days after intracranial inoculation, HER2-positive SKOV3-Luc-IP1 and HER2-positive MDA-MB-^231^Br tumor-bearing mice were injected intravenously with ^111^In-DTPA*-2Rs15d or ^111^In-DTPA*-trastuzumab. Outcomes of the trial showed that ^111^In-DTPA*-2Rs15d showed high tumor uptake in SKOV3.IP1 and MDA-MB-^231^Br tumor models (2.2 ± 0.4% IA/g and 4.52 ± 1.31% IA/g at 1 h p.i.). In addition, only very low accumulation in healthy tissue (<1% IA/g, except for kidney at 1 h p.i.) and fast renal clearance was observed. This was in contrast to the results obtained with ^111^In-DTPA*-trastuzumab. Here, only a low uptake of ^111^In-DTPA*-trastuzumab was observed in SKOV3.IP1 brain tumors. Thus, the study showed that the radiolabeled nanobody, in contrast to monoclonal antibodies, is able to pass the blood-brain barrier and is therefore the better option for molecular imaging of metastatic lesions in the brain [157].

In another trial, the first successful labeling of a nanobody with an α-emitter, Actinium-225, was described. For this purpose, the nanobody 2Rs15d was radiolabeled with ^225^Ac using the chelator p-SCN-Bn-DOTA. The nanobody ^225^Ac-DOTA-2Rs15d showed in vitro and in vivo a higher binding efficiency to HER2-overexpressing SKOV-3 cells than to low HER2-expressing MDA-MB-231 cells (4.01% ID/g vs. 0.49% ID/g after 2 h), indicating specific binding to the antigen and resulting in high tumor to normal tissue ratios. Moreover, coinjection of gelofusin reduced renal retention threefold, but in parallel a lower tumor uptake (4.01 ± 1.58% ID/g at 2 h p.i.) and a slightly higher liver retention (6.35% ID/g vs. 4.41% ID/g without gelofusin at 2 h p.i.) were observed in SKOV-3 tumor-bearing mice [158]. A study investigating the therapeutic efficacy of this radioconjugate for brain metastatic breast cancer showed that administration of ^225^Ac DOTA 2Rs15d alone or in combination with trastuzumab significantly increased the median survival in SKOV3.IP1 and MDA-MB-231Br brain tumor-bearing mice. In mice with intracranial SKOV3.IP1 tumors, the combined therapy even led to an extension of median survival by another 6.5 days compared to mice treated with ^225^Ac-DOTA-2Rs15d alone (29.5 days vs. 23 days). In addition, histopathological analyses showed no significant early toxicity, and renal retention was reduced by the simultaneous administration of 150 mg/kg gelofusin, making this radiolabeled nanobody a promising vehicle for targeted radionuclide therapy of HER2-positive brain lesions [157].

Choi et al. investigated the radiolabeling of the 5F7 nanobody with another α-emitting radionuclide, the halogen Astatine-211 (7.2 h). For this purpose, the nanobody was labeled with the two prosthetic agents [^211^At]-SAGMB or iso-[^211^At]-SAGMB and was evaluated in SCID mice with subcutaneous BT474M1 xenografts. Although the radiochemical yield, purity and in vivo behaviour with respect to nonspecific accumulation in spleen and lungs were similar for both radioconjugates, isomer-dependent differences in the in vivo stability of these nanobodies were observed. The iso-conjugate showed a higher tumor uptake and binding affinity to the HER2 antigen. In addition, it showed a higher tumor to background ratio and shorter renal retention than [^211^At]-SAGMB-5F7. Thus, iso-[^211^At]-SAGMB-5F7 proved to be the more promising and was further investigated in another study on ^211^At-labeled nanobodies [159]. In this study, the iso-conjugate was compared with two other precursors, m-MeATE and MSB, bound to the nanobody 2Rs15d. The [^211^At]-SAGMB-2Rs15d was found to be the preferred compound for targeted alpha therapy due to its high tumor uptake (8.90 ± 2.79% ID/g at 1 h p.i.), low background signals and rapid renal excretion. In addition, the other two nanobodies could be excluded from further studies due to high accumulation in the stomach, spleen and lungs and their low in vivo stability. After metabolisation and deastatination of the less stable radioconjugates, the free ^211^At is released back into the bloodstream, which leads to a high uptake in the aforementioned organs. Although the [^211^At]-SAGMB-2Rs15d showed high renal retention, this could be reduced by administration of gelofusin. This will be further investigated in a study on maximum tolerated dose, toxicity and therapeutic efficacy [160].

In a first attempt to label a nanobody with Lutetium-177, four different bifunctional chelators (p-SCN-Bn-DOTA, DOTA-NHS ester, CHX-A"-DTPA or 1B4M-DTPA) were compared to select the optimal chemical link between the radioisotope and a nanobody targeting HER2. Although high stability over time was achieved for all tested conjugates, the 2Rs15d conjugated with the 1B4M-DTPA chelator was found to be the best compound due to its high specific tumor uptake combined with the lowest background uptake [161]. In a subsequent study, this radioconjugate was investigated with a coinfusion of gelofusin in HER2-positive SKOV-3 tumor xenographted mice and compared to nanobodies with different C-terminal amino acid tag sequences (Myc-His-tagged, His-tagged and untagged). Between the four nanobodies investigated, the lowest renal retention was observed in the untagged ^177^Lu-DTPA-2Rs15d with simultaneous injection of 150 mg/kg gelofusin (6.52 ± 0.18% IA at 50 min p.i.). Also, specific tumor uptake (6.5 ± 0.2% IA/g at 1 h p.i.) and low background tissue and organ uptake (< 0.6% IA/g at 1 h p.i.) was observed for the untagged ^177^Lu-DTPA-2Rs15d with simultaneous injection of 150 mg/kg gelofusin. In a comparative study, ^177^Lu-DTPA-trastuzumab supplied a 6-fold higher dose to the tumor than the untagged ^177^Lu-DTPA nanobody. On the other hand, ^177^Lu-DTPA-trastuzumab showed a significant retention of radioactivity in the lung, liver, spleen, bone and blood. Nevertheless, no evidence of renal toxicity could be found in histological analyses and the administration of ^177^Lu-DTPA-2Rs15d led to an almost complete inhibition of tumor growth [137].

## Outlook

The many preclinical and clinical studies conducted in the recent years bear witness to the wide range of possible applications of antibody fragments such as affibodies and nanobodies, especially in the field of nuclear medicine. Their properties such as tissue permeability and rapid elimination from the blood make them ideal tools for targeted radiotherapy and molecular imaging. However, results of clinical studies show that nanobodies seem to be better suited for use as theranostatics in nuclear medicine due to their higher affinity to the target. One important point in theranostics is the choice of radionuclide. This should be stably linked to the nanobody and should not have a long half-life. The half-life of the radioisotope would then correlate with the short biological half-life of the nanobodies, thus avoiding high radiation exposure of the patients and allowing image acquisition within a couple of hours after application. In order to be able to use the therapeutic nanobodies in daily clinical practice, however, an appropriate blocker to protect the kidney from high radiation doses has to be researched beforehand or the nanobodies have to be modified in such a way that the renal retention is reduced. Not only the reduction of radiation exposure but also the reduction of toxicity in non-target tissues should be the aim of further investigations. The modification of nanobody to facilitate its passage through the blood-brain barrier is worth investigating, so that brain metastases can be better detected and treated in the future.

Introducing radiomics analyses of molecular imaging with radiolabelled HER2 targeting constructs (antibodies, affibodies or nanobodies) might further enhance the potential of this approach to support individualized management of breast cancer patients. Radiomic is defined as a set of methods for extracting and analysing quantitative data from biomedical images (features) to study individual tumor characteristics, clinical outcomes, and to develop computational models that can serve as tools for personalized diagnosis and treatment guidance [162].

## Conclusion

**Fig. 4:**
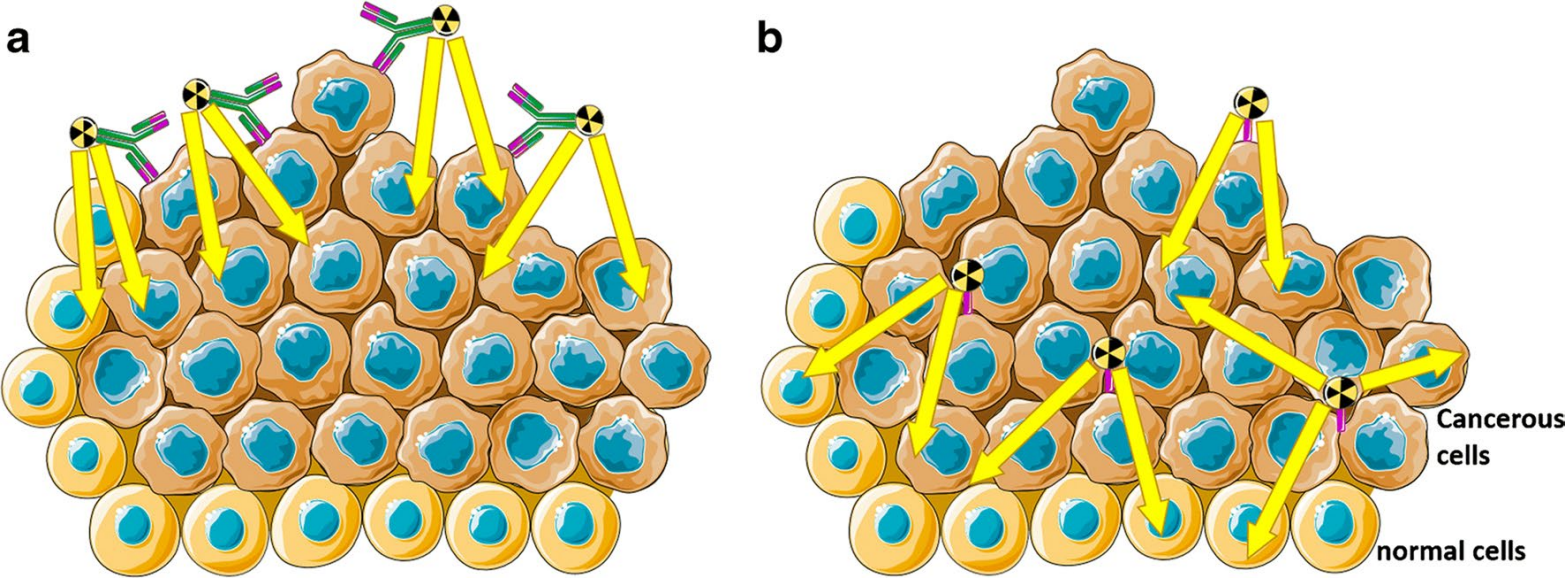
Schematic representation of tumor penetration of radiolabeled monoclonal antibodies (**a**) compared to radiolabeled nanobodies (**b**)

Monoclonal antibodies and antibody drug conjugates represent the preferred treatment options for HER2 positive breast cancers due to their high specificity and affinity to the antigen. In contrast to the in situ determination of HER2 expression, the use of radiolabeled antibodies in vivo allows the assessment of tumor heterogeneity, tumor accessibility and the use of molecular targeted therapies. However, the use of antibodies in molecular imaging is impaired by slow blood clearance, associated with slow and low tumor uptake and with limited tumor penetration potential. Nanobodies, on the other hand, are characterised by their low molecular weight, high stability, strong antigen-binding affinity, water solubility and their ability to penetrate deeper into the tumor than monoclonal antibodies do (Fig. 4) [163, 164]. These properties make them a preferable vehicle for molecular imaging as well radioimmunotherapy.

In addition, they can be administered intravenously, orally, intraperitoneally or intratumoral due to their chemical stability including the ability to withstand harsh conditions, chaotropic agents and pH extremes. Their rapid clearance from the organism is advantageous when applying in molecular imaging. As a result, even with short-lived radioisotopes, high-contrast images can be recorded within a few hours post injection, enabling early diagnosis and reduced radiation exposure of patients. In therapy, the small size radiolabeled nanobodies show themselves superior to the radiolabeled monoclonal antibodies due to their higher specificity and their ability to penetrate the tumor. On the other hand, the monoclonal antibodies could be preferred for therapeutic approaches due to their longer residence time in the blood plasma and the associated higher lethal radiation doses delivered to the tumor. However, the attempts to use nanobodies as vehicles are still ongoing and so far show promising results and they could be well preferred over their monoclonal counterpart in the field of targeted radionuclide therapy. Not unlikely further elaborated drug delivery approaches might contribute to the success of nanobodies.

## Abbreviations

ADC: Antibody drug conjugate
ADIBO: *N*-(3-aminopropionyl)-5,6-dihydro-11,12-didehydrodibenzo[b,f]azocine
ASCO: American Society of Clinical Oncology
AuNP: Gold nanoparticles
CAP: College of American Pathologists
CDR: Complementarity determining regions
CH2: Constant heavy 2
DAR: Drug to antibody ratio
DFO: Deferoxamine
DS8201: Trastuzumab deruxtecan
EMA: European Medicines Agency
ER: Estrogen
FDA: Food and Drug Administration
FISH: Fluorescence in situ hybridization
HcAb: Heavy chain antibody
HER2: Human epidermal growth factor receptor 2
IA: Injected activity
ID: Injected dose
IHC: Immunohistochemistry
MMAF: Monomethylauristatin F
NOTA: 1,4,7-triazacyclononane-1,4,7-triacetic acid
OS: Overall survival
PEG: Polyethylene glycol
PET: Positron emission tomography
PFS: Progression-free survival
PR: Progesteron
p-SCN-Bn-NOTA: S-2-(4-isothiocyanatobenzyl)-1,4,7-triazacyclononane-1,4,7-triacetic acid
RC-48: Hertuzumab Vedotin/Disitamab Vedotin
scFV: Single chain variable fragment
sdAb: Single domain antibody
SGMIB: *N*-succinimidyl 4-guanidinomethyl-3-iodobenzoate
SPAAC: Strain-promoted azide-alkyne cycloaddition (SPAAC)
SPECT: Single photon emission computed tomography
SYD985: [vic-] Trastuzumab Duocarmycin
T-DM1: Ado-trastuzumab emtansine
vc-*seco*-DUBA: Valine-citrulline-*seco* DUocarmycin hydroxyBenzamide-Azaindole
VHH: Variable domain of camelid heavy chain antibody
VNAR: Variable domain of the shark new antigen receptor
