# Correction to: HER2-directed antibodies, affibodies and nanobodies as drug-delivery vehicles in breast cancer with a specific focus on radioimmunotherapy and radioimmunoimaging

**DOI:** 10.1007/s00259-024-06792-w

**Published:** 2024-06-20

**Authors:** Betül Altunay, Agnieszka Morgenroth, Mohsen Beheshti, Andreas Vogg, Nicholas C. L. Wong, Hong Hoi Ting, Hans-Jürgen Biersack, Elmar Stickeler, Felix M. Mottaghy

**Affiliations:** 1 Department of Nuclear Medicine, University Hospital Aachen, RWTH Aachen University, 52074 Aachen, Germany; 2 Center of Integrated Oncology (CIO), Cologne and Düsseldorf, Universities of Aachen, Kerpener Str. 62, 50937 Bonn, Cologne, Germany; 3 Division of Molecular PET-Imaging and Theranostics, Paracelsus Medical University, 5020 Salzburg, Austria; 4 Nanomab Technology Limited, Shanghai, People’s Republic of China; 5 Department of Nuclear Medicine, University Hospital Bonn, Bonn, Germany; 6 Department of Gynecology and Obstetrics, RWTH Aachen, Aachen, Germany; 7 Department of Radiology and Nuclear Medicine, Maastricht University Medical Center (MUMC+), 6202 Maastricht, The Netherlands

**Correction to: European Journal of Nuclear Medicine and Molecular Imaging (2021) 48:1371–1389**

10.1007/s00259-020-05094-1

The authors regret that there are some errors in the original published article. The below article note should be removed.

*Betül Altunay and Agnieszka Morgenroth contributed equally to this work.*

On the other hand, the “**Authors’ contributions**” section should be corrected from:

Conceptualization, writing—original draft preparation BA, AM; writing—review and editing, all authors. All authors have read and agreed to the published version of the manuscript.

to:

Conceptualization, writing—original draft preparation BA; writing—review and editing, all authors. All authors have read and agreed to the published version of the manuscript.

The original article has been corrected.

